# Genetic Characterization of a 12th-Century Princely Burial in St. Sophia Cathedral of Veliky Novgorod

**DOI:** 10.32607/actanaturae.27986

**Published:** 2026

**Authors:** M. V. Leonova, I. K. Reshetova, K. V. Zhur, D. A. Vashunina, F. S. Sharko, N. G. Maksimov, M. V. Dobrovolskaya, N. A. Makarov, E. B. Prokhortchouk

**Affiliations:** Federal Research Center “Fundamentals of Biotechnology”, Russian Academy of Sciences, Moscow, 119071 Russia; Pirogov Russian National Research Medical University (RNRMU), Moscow, 117997 Russia; Institute of Archaeology, Russian Academy of Sciences, Moscow, 117292 Russia; National Research Center “Kurchatov Institute”, Moscow, 123182 Russia; Autonomous Non-Profit Organization for the Creation, Support and Development of the Historical and Cultural Electronic Encyclopedia and Library “RUNIVERS”, Moscow, 119071 Russia

**Keywords:** Rurikids, Mstislav Rostislavich the Brave, ancient DNA, whole-genome sequencing, IBD analysis

## Abstract

The necropolis of St. Sophia Cathedral in Veliky Novgorod, Russia, as noted in chronicle sources and cathedral inventories, contains the burials of five princes from the Rurikid dynasty and two princesses from their families. To clarify the genetic ancestry of two of the buried individuals, whose remains preserved the skeletal elements required for genetic analysis, DNA extracted from the remains was sequenced. Genetic analysis has revealed that the male individual from the sarcophagus near the southern wall of the Nativity side-chapel carries the Y-chromosomal haplogroup N1a and can be identified with high probability as Mstislav Rostislavich the Brave. Analysis of DNA extracted from female skeletal remains from the reliquary shrine in the Martyrius Porch revealed no close kinship with any of the known genomes of Rurikid dynasty members. A sculptural facial reconstruction based on the skull of Prince Mstislav Rostislavich and a digital facial approximation based on the cranium of the female individual were performed.

## INTRODUCTION

As a result of advances in Russian science and the development of laboratory infrastructure providing a full research cycle in historical genetics it has become possible to identify several key genetic characteristics of members of the ruling dynasty of Old Rus. Recent studies have enabled the reconstruction of genomes belonging to notable historical figures, including Prince Dmitry Alexandrovich, the son of Grand Prince Alexander Yaroslavich Nevsky [1], King Béla III of Hungary [2], and Béla Rostislavich, ruler of Mačva and Bosnia, the maternal grandson of King Béla IV of Hungary and the son of Rostislav Mikhailovich of the Rurikid dynasty [3]. All members of the Rurikid dynasty whose genomes have been published to date belong to Y-chromosome haplogroup N1a. Analysis of shared haplotypes confirms the biological kinship between these individuals. Recently published genetic data on Princess Maria Vsevolozha, wife of Grand Prince Vsevolod the Big Nest and great-grandmother of Dmitry Alexandrovich, have also contributed substantially to our understanding of the history of the princely lineage [1]. These data confirmed not only the Alan origin of Maria Vsevolozha herself, but also the absolute reliability of the identification of her remains and those of Dmitry Aleksandrovich. In turn, the established genetic connection between the Russian princes and the Árpád dynasty members provides conclusive evidence that the remains belong specifically to members of the Rurikid dynasty [3].

Further progress in the historical genetics of the medieval Rurikids was made possible by a new phase of work on the identification of remains from the necropolis of St. Sophia Cathedral in Veliky Novgorod, initiated by the Novgorod Metropolia, and carried out, in particular, in 2024. Samples for genetic analysis were taken from the skeletons of a male individual from the sarcophagus near the southern wall of the Nativity chapel and a female individual from the reliquary of the Martyrius Porch.

Chronicle records of the burials of five Rurikid princes in St. Sophia: Vladimir Yaroslavich, the eldest son of Yaroslav the Wise and builder of the cathedral (1052); Izyaslav Vladimirovich (1096); Mstislav Rostislavich the Eyeless (1178); Mstislav Rostislavich the Brave (1180); and Young Prince Vasily Mstislavich (1218). The charter granted to St. Sophia Cathedral in 1556 and the 17th-century cathedral inventories mention two female burials located in the princely tombs [4]. The remains of three princes and two women from princely families were exhumed from their original tombs between the 15th and 17th centuries, when their ecclesiastical veneration was established, and remained sacred relics of the St. Sophia pantheon until 1919. The formation of the St. Sophia necropolis as the principal pantheon of Veliky Novgorod was examined by V.L. Yanin, who reconstructed the original location of princely burials and the subsequent relocation of remains after canonization and demonstrated that the identification of certain burials when the veneration of Princes Vladimir Yaroslavich and Mstislav the Brave was established in 1439 had been erroneous [4]. Following 1919, the sarcophagi were opened, with the remains displayed in St. Sophia Cathedral, relocated during the war, and reinstated in the cathedral in 1944. Anthropologists and forensic medical professionals have repeatedly conducted expert evaluations of the remains from St. Sophia Cathedral [4, 5]. In 2016, at the request of the diocese, I.K. Reshetova described the remains and prepared a report titled “Anthropological Conclusion on the Study of Remains from the St. Sophia Cathedral,” which was submitted to the diocese and remains unpublished. For the most part, these conclusions are consistent with one another. The most detailed description of the remains was compiled by the anthropologist V.V. Ginzburg based on his examination in 1939 [5].

Samples from two “princely” burials lacking a documented archaeological context, P266♂ and P264♀, were submitted for genetic study. In church tradition, these remains are known as those of “Mstislav” and “Anna” the first wife of Yaroslav the Wise, who is not mentioned in the chronicles. Based on the comparative descriptions by V.V. Ginzburg and I.K. Reshetova, it can be concluded that the remains studied indeed originate from burials inside the cathedral. It was hypothesized that the remains with ID P266♂ could correspond to Mstislav Rostislavich the Eyeless (1178) or Prince Mstislav Rostislavich the Brave (1180), while the remains with ID P264♀ could belong to the wife of one of these princes. The genetic analysis was thus intended both to verify, in general terms, that the remains belong to members of the Rurikid dynasty, and to identify remains belonging to specific historical individuals buried in St. Sophia Cathedral.

## EXPERIMENTAL

Ancient DNA (aDNA) work was performed in a clean-room facility at the Federal Research Center “Fundamentals of Biotechnology” of the Russian Academy of Sciences (K.G. Skryabin Institute of Bioengineering). DNA was extracted from a tooth fragment (incisor), identification number P264♀, and an auditory ossicle (stapes), identification number P266♂, using a magnetic separation method [6] with Dabney extraction buffer and silica-coated magnetic beads (G-Biosciences, USA). DNA libraries for sequencing and estimation of the endogenous DNA content were prepared using the xGen™ ssDNA & Low-Input DNA Library Preparation Kit (Integrated DNA Technologies, USA). Additional libraries were subsequently prepared from the same DNA extract, pretreated with a mixture of uracil-DNA glycosylase (UDG) and endonuclease VIII (Endo VIII) [7]. The MyBaits Expert Human Affinities Prime Plus Kit (Daicel Arbor Biosciences, USA) was used for targeted genomic enrichment. Sequencing was performed on a HiSeq 1500 instrument (Illumina, USA) in 2 × 150 bp mode. Contaminant DNA reads were filtered via the BBDuk tool [8], with the output data processed in the PALEOMIX pipeline (version 1.2.14) [9], including adapter sequence trimming [10] and read alignment to the human reference genome (hg19/GRCh37) [11]. Quality filtering, indexing, sorting, and duplicate removal (rmdup) were performed using SAMtools (version 1.9) [12]. Genotypes were determined using the PileupCaller tool with the “--randomHaploid” option. Postmortem DNA damage patterns were analyzed using the MapDamage2 program [13]. The ADMIXTURE v.1.3.0 software [15] was used to evaluate the clustering of the sample under examination among the published ancient samples from the Allen Ancient DNA Resource (AADR) panel [14]. Single-nucleotide polymorphisms (SNPs) were pruned for sites in linkage disequilibrium using PLINK v.1.9 (--indep-pairwise 50 5 0.2) [16]. Principal component analysis (PCA) was performed using the smartpca tool from the EIGENSOFT package, using the sample set listed in *Supplementary Material 1*. Mitochondrial haplogroups were determined using the HaploGrep program [17]. Genomic modeling was performed using the qpWave and qpAdmix software programs (“allsnps: YES”). Genetic relatedness analysis was conducted using IBD-based methods [18] and READ [19]. The sex and age determination of the skeletal remains under study was conducted based on the morphological evaluation of the skull and postcranial skeleton, alongside an analysis of physiological stress markers and pathological conditions (*Supplementary Material 2*). The eye, hair, and skin colors were predicted using the HIrisPlex-S online tool [20]. The remains were radiocarbon-dated (*Supplementary Material 3*). Sequencing data for samples with the laboratory identification numbers P266♂ and P264♀ are available for download at http://russiangenome.ru/P264c.read1.fastq.bz2; http://russiangenome.ru/P264c.read2.fastq.bz2; http://russiangenome.ru/P266c.read1.fastq.bz2; http://russiangenome.ru/P266c.read2.fastq.bz2.

## RESULTS

**aDNA sequencing results**

The sequencing results of the DNA libraries prepared from the genetic material of the two individuals, the male (identification number P266♂) and the female (identification number P264♀), are presented in *Table 1* of *Supplementary Material 4*. The endogenous DNA content was 56.9% for P266♂ and 61.0% for P264♀ (*Supplementary Material 4, Fig. 1*). More than 57 and 28 million reads were generated, yielding 862,776 and 659,706 SNPs. Genetic sex determination [based on the ratio of reads mapped to the X and Y chromosomes] confirmed that P266♂ is male and P264♀ is female. Analysis of uniparental markers assigned P264♀ to mitochondrial haplogroup K1c1, and P266♂ to mitochondrial haplogroup H1b1 and Y-chromosome haplogroup N1a. Assessment of contamination levels revealed no detectable contamination in the analyzed samples (*Supplementary Material 4, Table 2*).

**Y-chromosome analysis of Rurikid dynasty members**

Phylogenetic analysis showed that the Y chromosomes of Prince Dmitry Alexandrovich, Béla Rostislavich, and sample P266♂ fall within the same clade and are highly similar to one another (*Supplementary Material 5*). Two medieval “Vikings” (VK504 and VK51, carrying Y-chromosomal haplogroup N1a1 (N-Z4908 and N-L550)) from present-day Estonia (Saaremaa Island) and Sweden (Gotland Island) occupy a close phylogenetic position relative to these samples. Close clustering was also observed with ancient individuals from Early Neolithic burial sites in the Cis-Baikal region − specifically Shamanka II (DA250, DA251, and DA362) and Lokomotiv (DA359), which carry the related N1a2 lineage − as well as an individual from Bolshoy Oleniy Island (BOO004.A0101, 2050–1500 BCE, N1a1/N-L1026). This placement of the ancient individuals is consistent with the known wide distribution of closely related lineages of this haplogroup across northern Eurasia from Siberia and the Cis-Baikal region to northeastern Europe and the Baltic.

**IBD analysis results**

The next step was an analysis of identity-by-descent (IBD) segments shared among the putative members of the Rurikid dynasty (*Fig. 1A*). This approach allows for verification of whether the skeletal remains from St. Sophia Cathedral belong to the Rurikid princely lineage. For this analysis, the genomes of Prince Dmitry Alexandrovich, King Béla III of Hungary, and Béla Rostislavich were imputed (statistical inference of missing genotypes) and then compared pairwise to identify shared IBD segments. The closer the degree of relationship reconstructed from the pedigree, the longer the IBD segments expected between the individuals compared. *Supplementary Material 6* shows the part of the Rurikid genealogical tree relevant to this study, which helps to interpret the hypotheses tested in terms of the kinship relationships between the DNA samples analyzed.

**Fig. 1:**
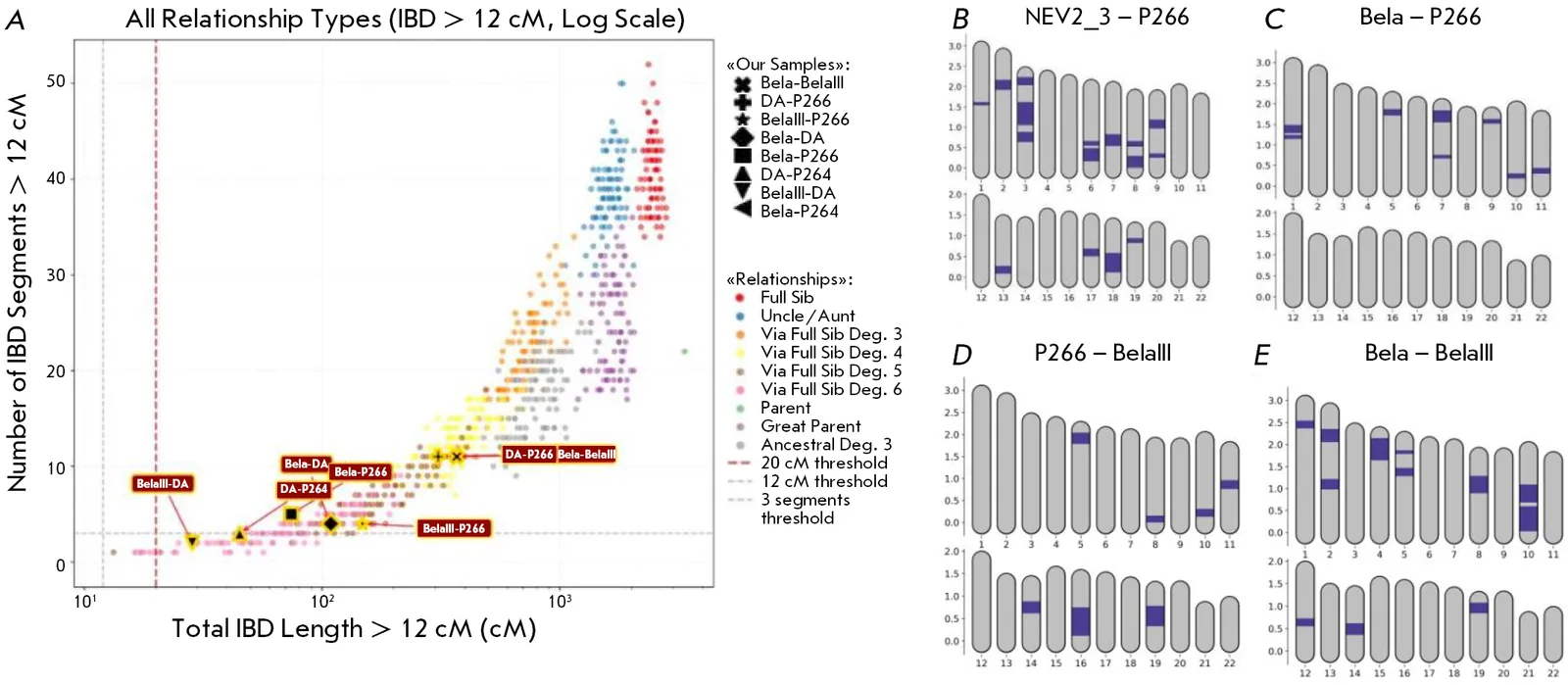
Degree of relationship relative to IBD segment matches (*A*). Pairwise comparison of IBD genomic segments between P266♂ and Dmitry Alexandrovich (NEV2_3) [1] (*B*), Béla Rostislavich (Béla) (*C*) [3], and King Béla III of Hungary (*D*) [2], alongside IBD sharing between Béla III and his great-great-grandson Béla Rostislavich (*E*)

The anthropological analysis (*Supplementary Material 2*) suggests that the skeletal remains under identification number P266♂ could belong to (i) Mstislav the Brave, (ii) Mstislav the Eyeless, or (iii) Vladimir Yaroslavich. For all three hypotheses, pairwise IBD comparison was carried out on the published Rurikid genomes (*Fig. 1A*), and for each hypothesis the mean absolute percentage error (MAPE) between the expected and observed total length of IBD segments ≥12 cM was calculated. The total of 322 cM obtained for the Béla III–Béla Rostislavich pair was used as the reference value for a fourth-degree relationship. The largest error was related to the hypothesis identifying the sample as Prince Vladimir Yaroslavich (MAPE = 79%), the error was somewhat lower for Mstislav the Eyeless (MAPE = 59%), while the identification as Mstislav the Brave showed the closest agreement with the expected values (MAPE = 15%).

The IBD analysis indicates a close genetic relationship between sample P266♂ and Dmitry Alexandrovich (total IBD ≥ 12 cM = 318 cM), comparable to that between King Béla III of Hungary and his great-great-grandson Béla Rostislavich (total IBD > ≥ 12 cM = 322 cM) (*Fig. 1E*). The IBD sharing profile for sample P266♂ is more consistent with the expected position of Prince Mstislav Rostislavich the Brave than with Mstislav Rostislavich the Eyeless. According to the pedigree, Mstislav Rostislavich the Brave is connected to Dmitry Alexandrovich through the maternal lineage of Alexander Nevsky and represents a great-great-grandfather to Dmitry Alexandrovich.

Further argument supporting the assignment of the skeletal remains to Prince Mstislav Rostislavich the Brave is the high degree of genetic affinity between the P266♂ genome and the genome of King Béla III of Hungary (189 cM), whereas the total length of IBD segments ≥12 cM shared with Béla Rostislavich was 62 cM. Prince Mstislav Rostislavich the Brave is expected to be genealogically closer to Béla III, since both descend from a common ancestor within two generations – Prince Mstislav I the Great. Prince Mstislav the Brave is his grandson through Rostislav Mstislavich, and Béla III is his grandson through his daughter Euphrosyne of Kiev. However, if the relatedness between P266♂ and Béla Rostislavich were due solely to the line of Béla III, approximately 12 cM would be expected, whereas 62 cM is observed. The additional segments could have been inherited by Béla Rostislavich through the line of his father Rostislav Mikhailovich, provided that P266♂ is Mstislav the Brave. Conversely, if the P266♂ genome belonged to Mstislav the Eyeless, the established closeness to both Dmitry Alexandrovich and Béla III would be harder to reconcile with the observed values. The IBD segments between the P266♂ genome and the Béla Rostislavich genome, as well as between the P266♂ genome and the Béla III genome, are shown in *Fig. 1C,D*.

The IBD analysis revealed no close genetic relatedness between sample P264♀ and any of the known Rurikid genomes, or with sample P266♂ (*Supplementary Material 7, Fig. 1A,B,C*). The hypothesis based on church tradition, that P264♀ might be Anna of Novgorod, the wife of Yaroslav the Wise, remains unconfirmed. Furthermore, genetic connections were examined between sample P264♀ and Western Slavs, whose genomes have recently been published [21]. In the “Gesta Hammaburgensis Ecclesiae Pontificum” Adam of Bremen reports that Olof, the “most Christian king of the Swedes,” married a Slavic woman of the Obotrites named Estrid, who gave birth to Jacob and Ingegerd, the latter subsequently becoming the wife of Yaroslav [22]. Imputation and pairwise IBD analysis were carried out for sample P264♀ and 129 Slavic genomes representing populations to which the Obotrites may have belonged. No West Slavic genome shared an IBD segment longer than 8 cM with P264♀ (*Supplementary Material 8*), indicating the absence of even distant kinship. In addition, Zenczak M. et al. [23] recently studied skeletal remains attributed to Bolesław III, the Wrymouth of the Piast dynasty. According to historical sources, Bolesław III was a great-great-grandson of Ingegerd in the direct maternal line and carried mitochondrial haplogroup T2b4b.

The genetic ancestry of the individuals studied was assessed by principal component analysis (PCA) and ADMIXTURE analysis.

**Results of PCA**

The plot of the first two principal components (PC1 and PC2) demonstrates that the genomes of P266♂, P264♀, King Béla III of Hungary, and Béla Rostislavich cluster within the cluster of medieval European individuals from the territories of modern Finland, Denmark, Sweden, Norway, and Russia. For example, next to specimen P266♂ is a medieval Slavic specimen from Vladimir (Sungir 6). This position clearly separates them from the genomes of Prince Dmitry Alexandrovich and his great-grandmother Maria Vsevolozha, which plot closer to ancient samples from the steppe and the Caucasus, respectively (*Fig. 2*).

**Fig. 2:**
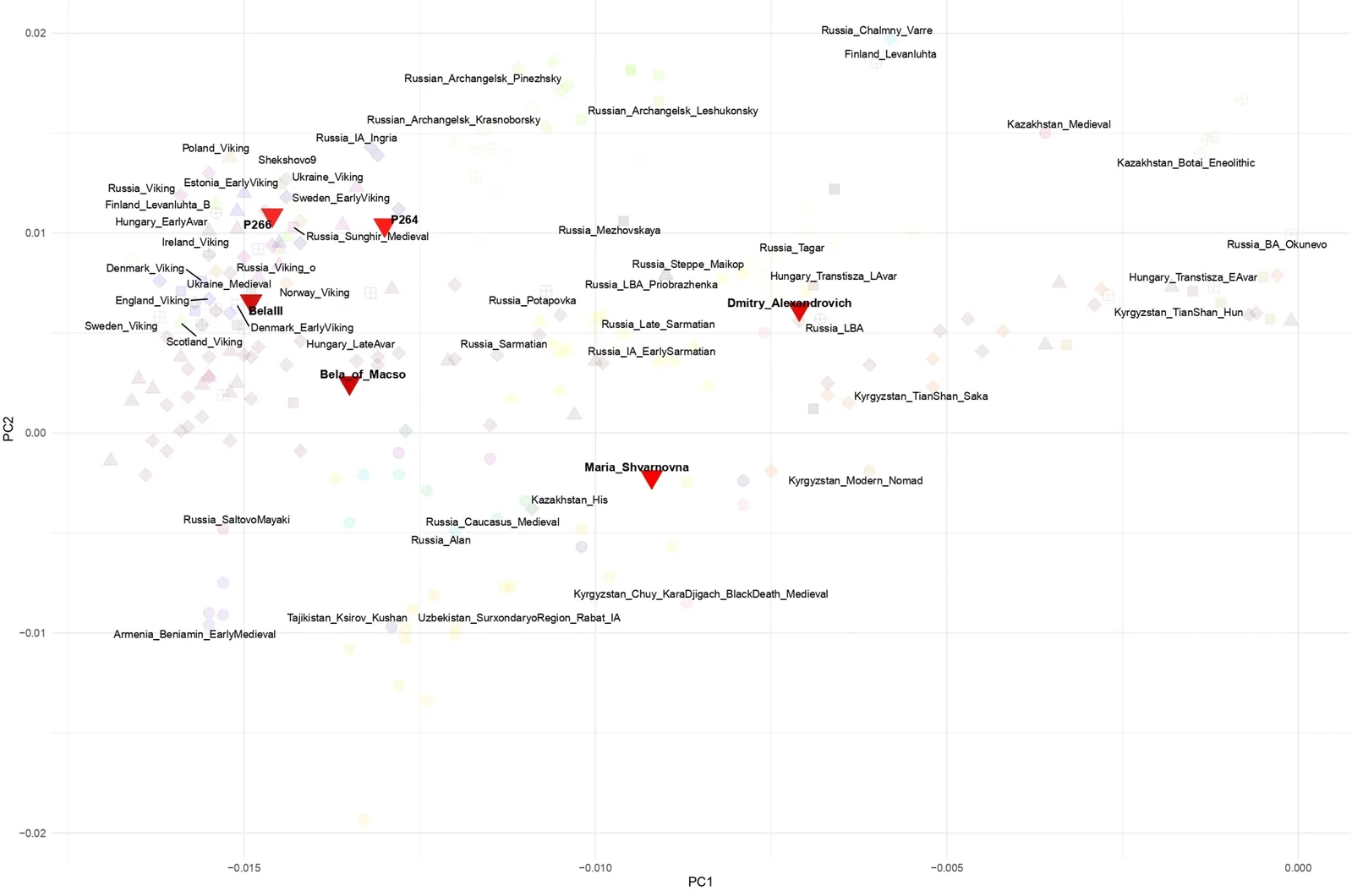
Principal component analysis (PCA) results for samples P264♀ and P266♂, projected onto previously published genomes of Rurikid [1] and Árpád [2, 3] representatives, as well as genomes from ancient populations

PCA with the projection of Early Medieval and Medieval Slavs from the study by Gretzinger et al. [21] demonstrated that female individual P264♀ can be assigned to the pan-Slavic medieval cluster, whereas male individual P266♂ falls within the “Viking” cluster (*Supplementary Material 9*).

Similar results were obtained when projecting the samples onto a PCA of modern populations (*Fig. 3*). Sample P266♂ was found to be closest to genomes from Norway and Iceland, while the P264♀ genome plots closest to samples from south-central Russia (Kursk and Belgorod).

**Fig. 3:**
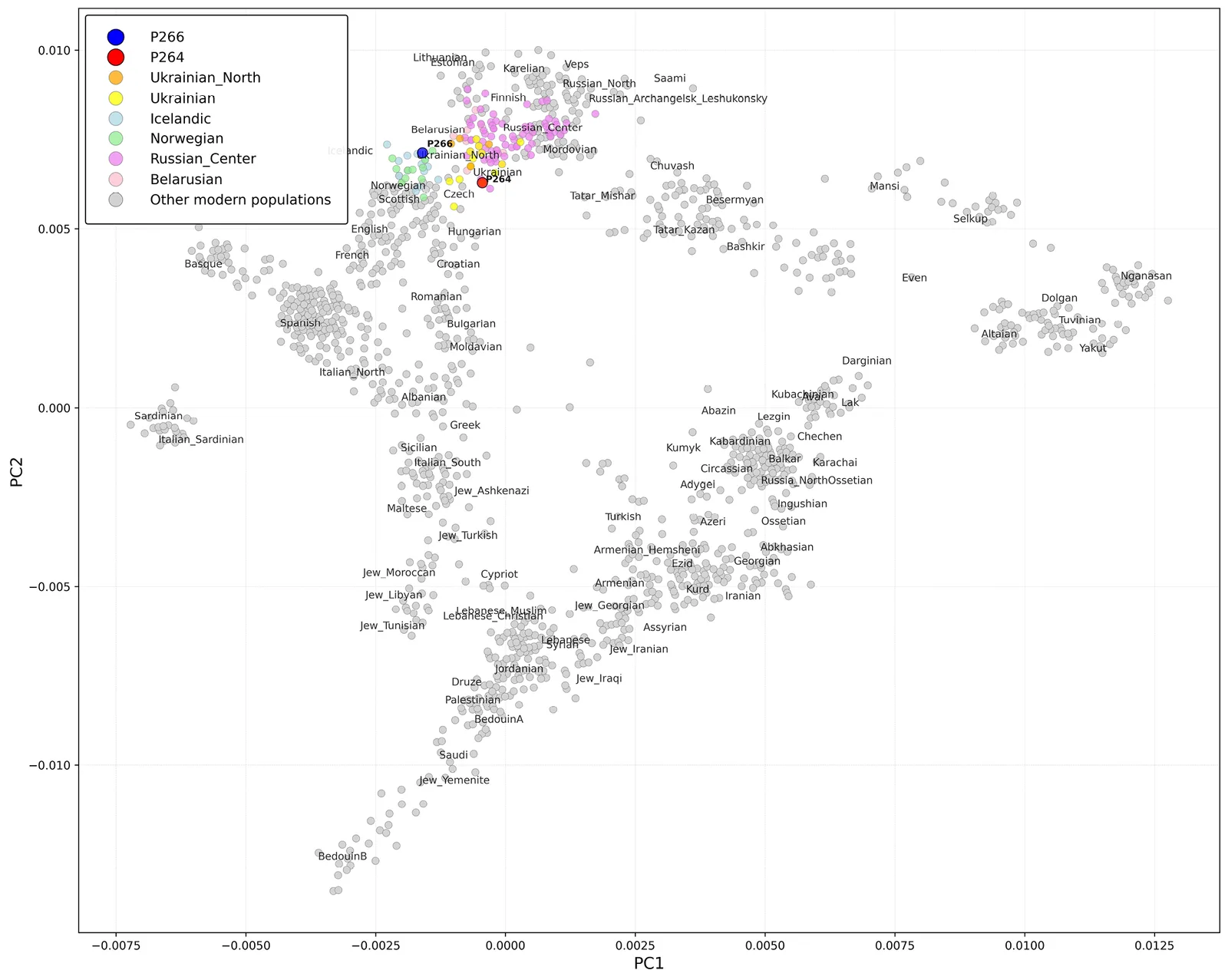
Principal component analysis (PCA) of individuals P264♀ and P266♂, projected onto present-day populations

**ADMIXTURE results**

*Figure 4* presents the ancestral component decomposition (K = 5) of the genomes of the individuals under analysis, as well as the genomes of representatives from medieval Viking Age populations across modern-day Finland, Denmark, Sweden, Norway, and Russia [24]. For a more detailed breakdown of the decomposition results at K = 4–6, the reader is referred to *Supplementary Material 10*.

**Fig. 4:**
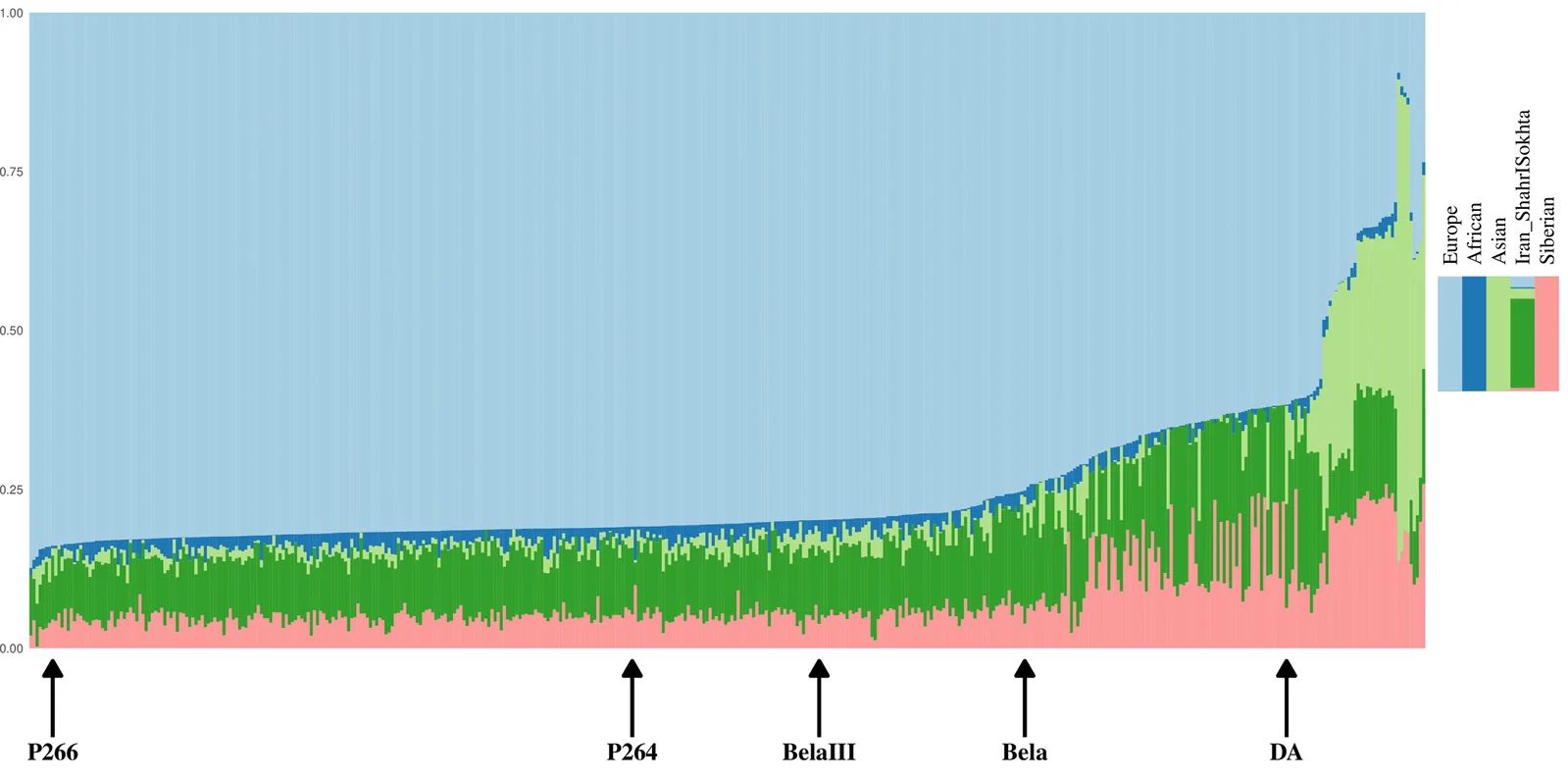
Ancestral component decomposition (K = 5) of the genomes under analysis

The decomposition results indicate that when the samples are sorted by the proportion of the putatively European component K1 (which is most pronounced in the Bulgaria_N sample), individual P266♂ falls at the beginning of the distributed sequence. Individual P264♀ and Béla Rostislavich are positioned in the middle of this sequence. In contrast, Prince Dmitry Alexandrovich, as previously established, exhibits a significantly higher proportion of Asian and Siberian components and accordingly the lowest proportion of K1, placing him at the opposite end of the series (*Supplementary Material 10*).

**Results of F4-statistics**

Chronicle data indicate that the grandmother of Prince Mstislav Rostislavich the Eyeless and wife of Prince Yuri Dolgorukiy was the daughter of the Cuman Khan Aepa. (The Laurentian and Ipatiev chronicles document the marriage of Prince Yuri Dolgorukiy, also known as Prince Girgen, to the Khan’s daughter on January 12, 1108.) These findings suggest a notably substantial steppe, Asian genetic influence in the P266♂ genome, assuming it belongs to Prince Mstislav Rostislavich the Eyeless. Despite the absence of steppe ancestry indicators in sample P266♂ during previous analytical stages, the hypothesis attributing the P266♂ genome to Prince Mstislav Rostislavich the Eyeless was further tested by calculating the F4-statistics in the configuration (Yoruba, Test, C, D), with Test representing one of the East or Central Asian populations, C representing various medieval Viking Age populations, and D representing the P266♂ genome analyzed. Significantly positive statistic values would indicate gene flow from East and Central Asian populations into the sample under analysis. According to the results obtained, no East or Central Asian population yielded significantly positive F4-statistic values for the P266♂ genome (Z-score >= 3) (*Fig. 5A* and *Supplementary Material 11*).

**Fig. 5:**
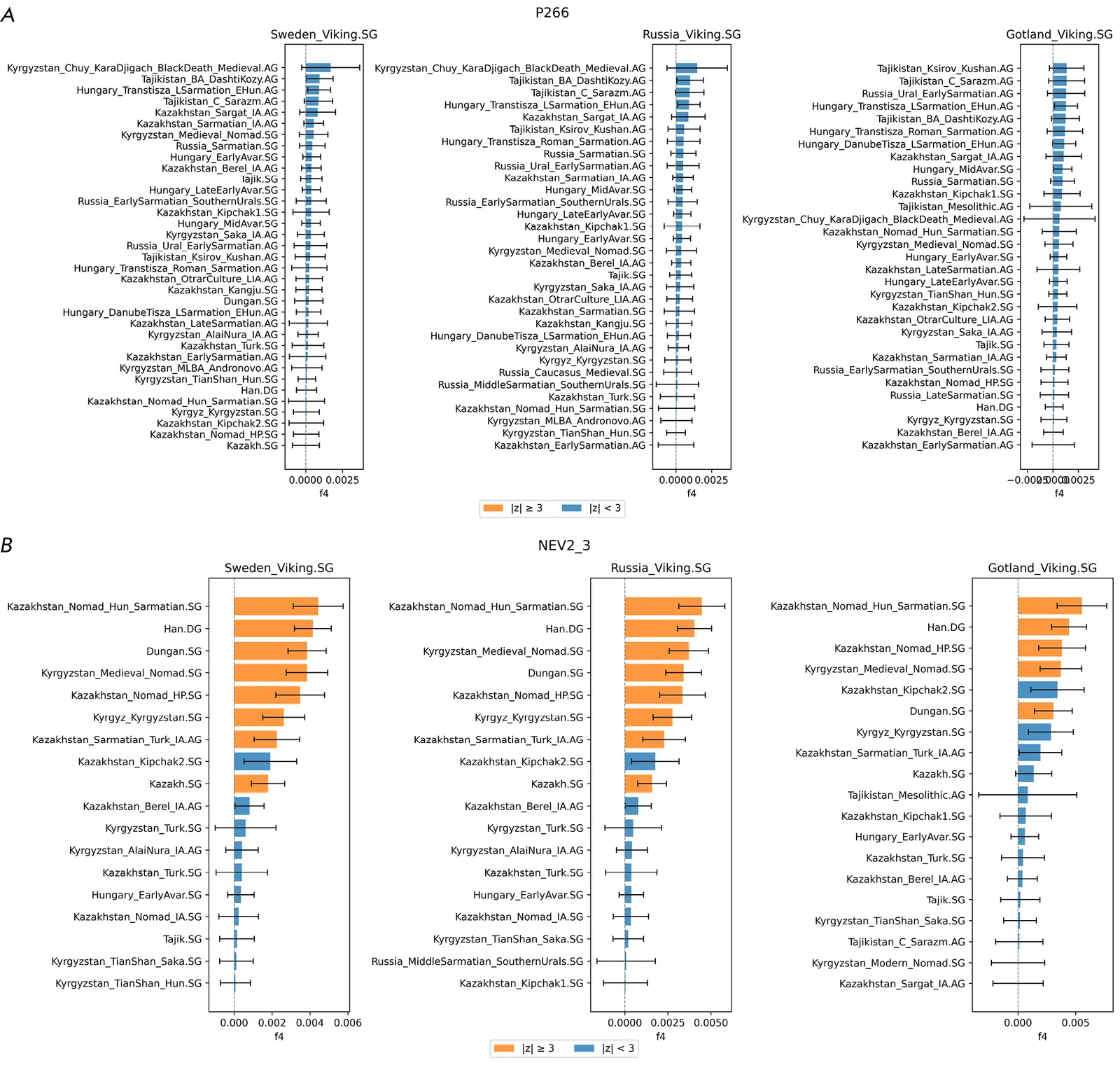
F4-statistic values for the configuration (Yoruba, Test, C, D). (*A*) D = P266♂ and (*B*) D = NEV2_3 [1], with three reference populations C: Sweden_Viking.SG [24], Russia_Viking.SG [24], and Gotland_Viking.SG [26], and steppe and Asian populations as Test. Values with Z-scores ≥ 3 are considered statistically significant

At the same time, significantly positive values were obtained for the genome of Prince Dmitry Alexandrovich in an analogous F4-statistics configuration (*Fig. 5B*) when using populations such as Kazakhstan_Nomad_Hun_Sarmatian, Han, Dungan, Kirgizstan_Medieval_Nomad, Kazakhstan Nomad, Kyrgyz_Kyrgyzstan, Kazakhstan_Sarmatian_Turk, and Kazakh as Test, provided that medieval Viking Age populations from modern-day Sweden and Russia (Russian_Viking, Sweden_Viking) were used as C. Statistically significant positive F4-statistic values were also obtained when using the Gotland_Viking population, but only in combination with East and Central Asian populations such as Kazakhstan_Nomad_Hun_Sarmatian, Han, Dungan, Kirgizstan_Medieval_Nomad, and Kazakhstan Nomad.

These results indicate that the genome of individual P266♂ lacks evidence of further gene flow from East and Central Asian groups. The genome of Prince Dmitry Alexandrovich (Nev2_3), which was used as a reference for this method and is known to have a steppe component, demonstrated positive F4-statistic values in a similar configuration. Accordingly, it is reasonable to conclude that the P266♂ genome most likely belongs to Mstislav Rostislavich the Brave, whose direct ancestors are not reported in chronicle sources to have steppe or Asian origins.

**IBD clustering results**

The close position of the sample attributed to Mstislav the Brave (P266♂) to the “Vikings” in the PCA and ADMIXTURE analyses could reflect kinship between members of the Rurikid dynasty and medieval Scandinavian populations, which would be consistent with the Normanist theory. To test this hypothesis, we imputed the genomes of 392 previously published “Vikings” [24] for subsequent pairwise IBD analysis (*Supplementary Material 12*). Due to the limitations of the method used [18], only genomes from the “Vikings” series with more than 600,000 SNPs from the 1,240k panel were used for interpretation. Pairs sharing more than 12 cM were used to construct the IBD network using the ForceAtlas layout and Leiden clustering. *Figure 6* visualizes the IBD connections within the largest Viking cluster, including representatives of the Rurikid dynasty, alongside the genetically related Béla III and Béla Rostislavich.

**Fig. 6:**
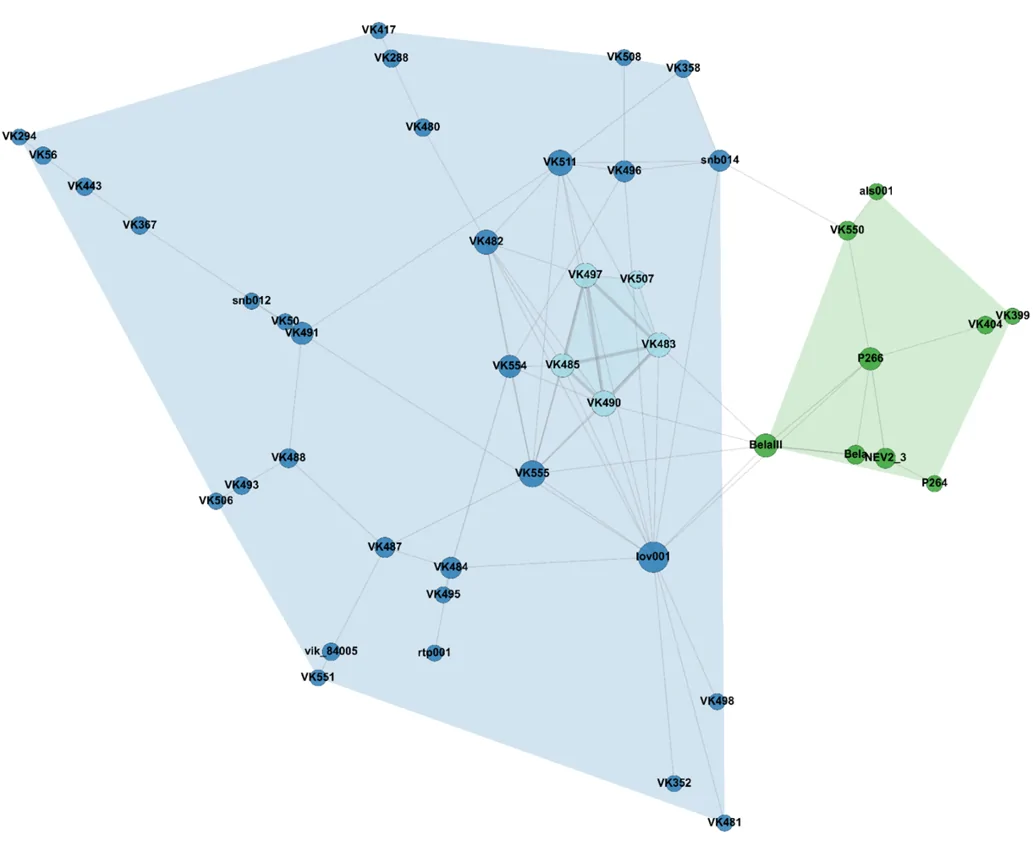
Clustering based on IBD connections with segment lengths > 12 cM

Four representatives of the Rurikid and Arpad dynasties (NEV2_3, P266♂, Béla, and Béla III) and sample P264♀ cluster together with two Swedish “Viking” samples, VK404 and VK399 (green cluster), whose remains were excavated in Varnhem (900–1200 CE, Varnhem, Sweden), as well as VK550 (700–800 CE, Salme, Estonia) and als001 (575–600 CE, Alsike, Sweden). Individual IBD connections for P266♂ with the “Vikings” are restricted to VK550 (18 cM), lov001 (17 cM), and VK404 (15 cM) (*Supplementary Material 12*). The Rurikid cluster, via Mstislav the Brave (P266♂) and Béla III, is linked to an extensive blue “Viking” cluster from Estonia and Sweden. This connection is primarily mediated through a female individual from the early “Viking” group, lov001 (sharing a single 17 cM segment on chromosome 14 common to all three targets), who lived during the Vendel Period and was buried approximately between 475 and 525 CE in Lovö near Stockholm. This individual also boasts the largest number of IBD links to other “Vikings,” which may indicate a substantial genetic contribution to the population. The fact that P266♂ and Béla III share the same segment with lov001 may suggest that they inherited it from a common ancestor. Sample P264♀ falls into the same green cluster solely because of a single link to NEV2_3 (38 cM) and has no IBD links to the “Vikings” of its own.

**Anthropological reconstruction results**

A sculptural facial reconstruction of the presumed Prince Mstislav Rostislavich the Brave was produced using the method of M.M. Gerasimov (reconstruction by I.K. Reshetova), and a digital craniofacial approximation was carried out based on the skull of the female individual P264♀ (approximation by A.I. Nechvaloda) (*Fig. 7*). SNPs informative for pigmentation were recovered in samples P264♀ and P266♂, allowing hair, skin, and eye color to be predicted. Blue eyes were predicted with the highest probability for both individuals (*P* = 0.911 and *P* = 0.932, respectively), as was [blond] hair (*P* = 0.676 and *P* = 0.738). For skin color, the [pale] and [intermediate] categories received similar probabilities (0.484 and 0.489 for P264♀; 0.528 and 0.439 for P266♂), so the individuals cannot be assigned unambiguously to either category (*Supplementary Material 13*). The skull of individual P266♂ can be characterized as mesodolichocranic, high (approaching very high values), with a relatively narrow face, a well-defined horizontal profile of the midface, and strongly projecting of the nasal bones. The cranium is distinguished by its large overall dimensions and robusticity. These morphological characteristics indicate the Caucasoid appearance of the individual. When combined with data about light-colored eyes, skin, and fair hair, these morphological characteristics confirm the expected Northern European appearance of the members of the Rurikid dynasty. The female skull is distinguished by dolichocranic, a relatively low vault, large overall dimensions for a female, a well-defined horizontal facial profile, and significantly protruding nasal bones. Emphasis should be placed on light pigmentation of the eyes, hair, and skin, as demonstrated by genetic methods and visualized in a reconstruction created using digital approximation. Consequently, the young woman has been determined to exhibit a complex of traits typical of medieval Northern European populations.

**Fig. 7:**
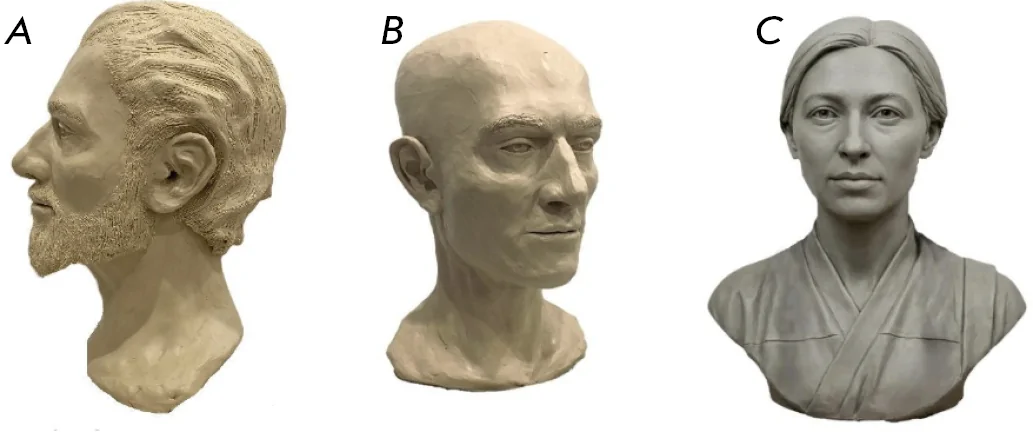
Anthropological reconstruction. (*A, B*) – Sculptural portrait of the presumed Prince Mstislav Rostislavich the Brave (reconstruction by I. K. Reshetova); (*C*) – Digital approximation based on the female cranium (digital approximation by A. I. Nechvaloda)

## DISCUSSION

The study of the remains from St. Sophia Cathedral in Novgorod provides additional genetic data for the genetic characterization of the Rurikid dynasty. The paleogenetic analysis indicates that the skeletal remains P266♂ most probably belong to Mstislav Rostislavich the Brave (†1180). Mstislav the Brave was the great-grandson of Vladimir Monomakh, grandson of Mstislav Vladimirovich, heir to the Kiev throne, and son of Rostislav Mstislavich, Prince of Smolensk, who was invited to the Kiev throne in 1159 and held it until his death in 1167. Mstislav was born in the late 1140s or early 1150s and was first mentioned in the chronicles under the year 1161. Throughout most of his life, Mstislav Rostislavich fought for the South Russian princely thrones and Smolensk, having minimal involvement with Novgorod, where he was invited to rule in 1179 [4, 27]. Marked by military conflicts and the rivalry between princely coalitions, this life has much in common with numerous other princely biographies from the 12th century. The designation “the Brave,” applied to Mstislav, is first documented in the catalogue of Novgorod princes found in the First Novgorod Chronicle of the Younger Recension, compiled during the mid-15th century [27, 28]. Of all the Rurikid remains studied genetically to date, those of Mstislav Rostislavich are the earliest, representing one of the genetic profiles of the third quarter of the 12th century. Significantly, this profile lacks the steppe component that is distinctly apparent in the genetic profile of Dmitry Alexandrovich, who represents the Rurikids of the second half of the 13th century. This finding is consistent with established knowledge regarding the nature of the association between the Rus’ ruling elite and the Polovtsian political entities during the late 12th and early 13th centuries. This was a period when marital alliances between Russian princes and women from Cuman princely lineages became particularly widespread [29].

The skeletal remains of P264♀ belong to a female individual associated with the East Slavic genetic cluster, distinct from the Rurikid princes, whose genetic profiles have been established by recent research. V.L. Yanin reasonably argued that the female remains discovered within princely tombs presumably belong to the wives of the princes [4]. If the skeletal remains, identified in ecclesiastical tradition as the remains of “Anna,” were originally placed in the tomb of Mstislav Rostislavich the Brave (rather than that of Vladimir Yaroslavich), it is reasonable to conclude that they belong to the wife of Mstislav the Brave, the daughter of Prince Gleb Rostislavich of Ryazan, whose name has not been preserved. However, given the historically documented relocation of the remains, one cannot exclude the possibility that they belonged to the wife of the other Mstislav, namely Mstislav Rostislavich the Eyeless, who was the daughter of Yakun Miroslavich, the Novgorod Posadnik [4].

## Abbreviations

aDNA: ancient DNA
SNP: single nucleotide polymorphism
PCA: principal component analysis
